# Detecting inversions with PCA in the presence of population structure

**DOI:** 10.1371/journal.pone.0240429

**Published:** 2020-10-29

**Authors:** Ronald J. Nowling, Krystal R. Manke, Scott J. Emrich

**Affiliations:** 1 Electrical Engineering and Computer Science, Milwaukee School of Engineering, Milwaukee, WI; 2 Physics and Chemistry, Milwaukee School of Engineering, Milwaukee, WI; 3 Electrical Engineering and Computer Science, University of Tennessee–Knoxville, Knoxville, TN; Chuo University, JAPAN

## Abstract

Chromosomal inversions can lead to reproductive isolation and adaptation in insects such as *Drosophila melanogaster* and the non-model malaria vector *Anopheles gambiae*. Inversions can be detected and characterized using principal component analysis (PCA) of single nucleotide polymorphisms (SNPs). To aid in developing such methods, we formed a new benchmark derived from three publicly-available insect data. We then used this benchmark to perform an extended validation of our software for inversion analysis (Asaph). Through that process, we identified and characterized several problematic test cases liable to misinterpretation that can help guide PCA-based inversion detection. Lastly, we re-analyzed the 2R chromosome arm of 150 *An. gambiae* and *coluzzii* samples and observed two inversions (2Rc and 2Rd) that were previously known but not annotated in these particular individuals. The resulting benchmark data set and methods will be useful for future inversion detection based solely on SNP data.

## Introduction

Chromosomal inversions play an important role in ecological adaptation by enabling the accumulation of beneficial alleles [1–4] and, in some cases, lead to reproductive isolation [5]. For example, the 2La inversion in the *Anopheles gambiae* mosquito complex has been associated with thermal tolerance of larvae [6], enhanced desiccation resistance in adult mosquitoes [7, 8], and susceptibility to at least one species of malaria parasite (*Plasmodium falciparum*) [9]. Inversions enable multiple mutually-exclusive traits to be maintained in the same population; inversion genotype frequencies and expressions of traits can vary seasonally [10] or spatially [6, 7, 11].

Principal component analysis (PCA) of single-nucleotide polymorphism (SNP) data is particularly attractive for detecting inversions (see Table 7 for a comparison of existing software). Inversions accumulate mutations private to each inversion orientation; these mutations are inherited by offspring but not shared across different orientations due to reduced recombination in the inversion region. For large inversions, the number of mutations correlated with each inversion form can be quite substantial and generate a large signal detectable by PCA [12–14]. Samples tend to cluster by their inversion genotypes, enabling inference of genotypes with clustering algorithms (e.g., K-Means or Gaussian Mixture Models) [15]. PCA-based methods successfully detected inversions in a number of organisms including insects (*Anopheles* mosquitoes [1, 16, 17]), fish (Atlantic cod [18–24]), birds (zebra finches [25] and great tits [26]), and plants (sunflowers [27]).

PCA of SNP data has wide-ranging uses in population genetics beyond inversion detection. PCA has been used to visualize relationships between samples [28], correct for stratification in genome-wide association studies (GWAS) [29], and as a pre-processing step for inferring population structure with clustering algorithms [30, 31]. Multiple phenomena including inversions and populations structure induce clustering in PCA scatter plots. Clusters can be mischaracterized if care is not taken to set up experiments appropriately (e.g., ensure samples are drawn from a single geographic region and population).

Inspection and visualization of the SNPs associated with principal components (PCs) or clusters enables more precise inversion detection and allows for inversion detection even when population structure is present. SNPs captured by a principal component can be identified by inspecting the loading factors [32, 33], association testing with PC coordinates or cluster IDs [16, 34–36], or analysis of variance using population genetics statistics such as *F*_*ST*_ [27]. When SNP association values are plotted along a chromosome (e.g., Manhattan plots), inversion regions stand out due to the presence of a step-function like pattern with a large number of associated SNPs in the inverted region and few outside of the region.

We gathered and curated the publicly-available SNP data sets from the *Drosophila* Genetic Reference Panel v2 (DGRP2) [37, 38], 1000 *Anopheles* Genomes project [17], and 16 *Anopheles* Genomes project [39] to create a benchmark for inversion analysis methods. Samples in these data sets had been experimentally genotyped for several well-studied large inversions in their original papers. These data provided interesting test cases such as complex relationships between inversions genotypes and population structure (the *Anopheles* samples) and each other (e.g., inversions of the 3R chromosome arm of the *D. melanogaster* samples). These data are important as many insects (including medically-important vectors and agricultural pests) do not have large, polytene chromosomes and must be analyzed with computational inversion detection techniques.

Using this new benchmark, we validated our inversion detection framework Asaph [16]. In our original paper, we only evaluated our framework on the 34 *Anopheles gambiae* and *coluzzii* samples from the 16 *Anopheles* Genomes project [39]. We demonstrated the value of this framework to the biological community by characterizing inversions on the 2R chromosome arm of 150 *Anopheles* samples from Burkina Faso [17]. We detected the presence of the 2Rc and 2Rd inversions in the *An. coluzzii* samples.

## Materials and methods

### Formation of three test sets

We constructed three test sets from publicly-available insect population genomics data [17, 37–39]. We downloaded the VCF files from the *Drosophila* Genetic Reference Panel v2 [37, 38] project web site, for the phase 1 AR3 data release from the 1000 *Anopheles* Genomes [17] project web site, and for 16 *Anopheles* genomes from project from the Dryad Digital Repository [40]. Sample IDs and inversion genotype annotations either came from the supplemental materials of the papers [37–39] or the 1000 *Anopheles* Genomes project web site. VCFTools [41] was used to create a separate VCF file for each chromosome arm and select biallelic SNPs. We performed PCA of SNPs from across the entire *Drosophila* genome; sevens samples (lines 348, 350, 358, 385, 392, 395, and 399) appeared to be outliers and were removed. For the 1000 *Anopheles* genomes project data, we selected the 150 *An. gambiae* and *coluzzii* samples from Burkina Faso. We previously selected 34 *An. gambiae* and *coluzzii* samples from Burkina Faso, Cameroon, Mali, and Tanzania from the 16 *Anopheles* genomes project data. The *Drosophila* 3L chromosome arm contained several low-frequency inversions (*In(3L)P*, *In(3L)M*, and *In(3L)Y*) [37, 38], so we filtered out the inverted individuals (lines 31, 69, 136, 426, 721, and 913) to allow 3L to be used in the negative test set. We also created a VCF file of the 2L SNPs from only the 81 Burkina Faso *An. gambiae* samples from the 1000 *Anopheles* Genomes data sets. We calculated inversion frequencies as (2 * homo. inv. samples + hetero. samples)/(2 * samples).

We provided scripts and inversion genotype labels for the benchmark data set in our public GitHub repository (https://github.com/rnowling/asaph). A provided script implements the steps above for processing data from the original repositories (provided by the user). Citations are provided in the documentation to aid users in citing the original papers.

### Methods for detecting, genotyping, and localization of inversions from SNP data

We compared three overlapping PCA-based methods (Scatter plots from PCA with clustering, PC-SNP association tests, and cluster-SNP association tests) for analysis of inversions using SNP data. The three methods differ in their capabilities (e.g., genotyping and localization) and sensitivity to parameters (e.g., selecting PCs and number of clusters) (see Table 1). All three methods are able to detect inversions but with different levels of precision. Inversions can be localized using either form of association testing, but only clustering can infer genotypes. Inversion detection was easier with the PC-SNP association tests since the cluster-SNP association tests were sensitive to using the correct combination of PCs and number of clusters.

**Table 1:** Comparison of methods. The capabilities of three PCA-based methods (PCA scatter plots with optional clustering and association testing SNPs against either cluster labels or PC coordinates) are summarized. We compare the methods on detecting, genotyping, and localizing inversions in terms of capability, easy of use, and potential for ambiguous results.

	PCA Scatter Plots	Clustering	Cluster-SNP Association Tests	PC-SNPAssociation Tests
**Detects Inversions**	Yes	Yes	Yes	Yes
**Infers Inversion Genotypes**	No	Yes	No	No
**Localizes Inversions**	No	No	Yes	Yes
**Ease of Use**	Easy	Moderate	Difficult	Easy
**Potential for Ambiguous Interpretation**	Yes	Yes	No	No

**Fig 1:**
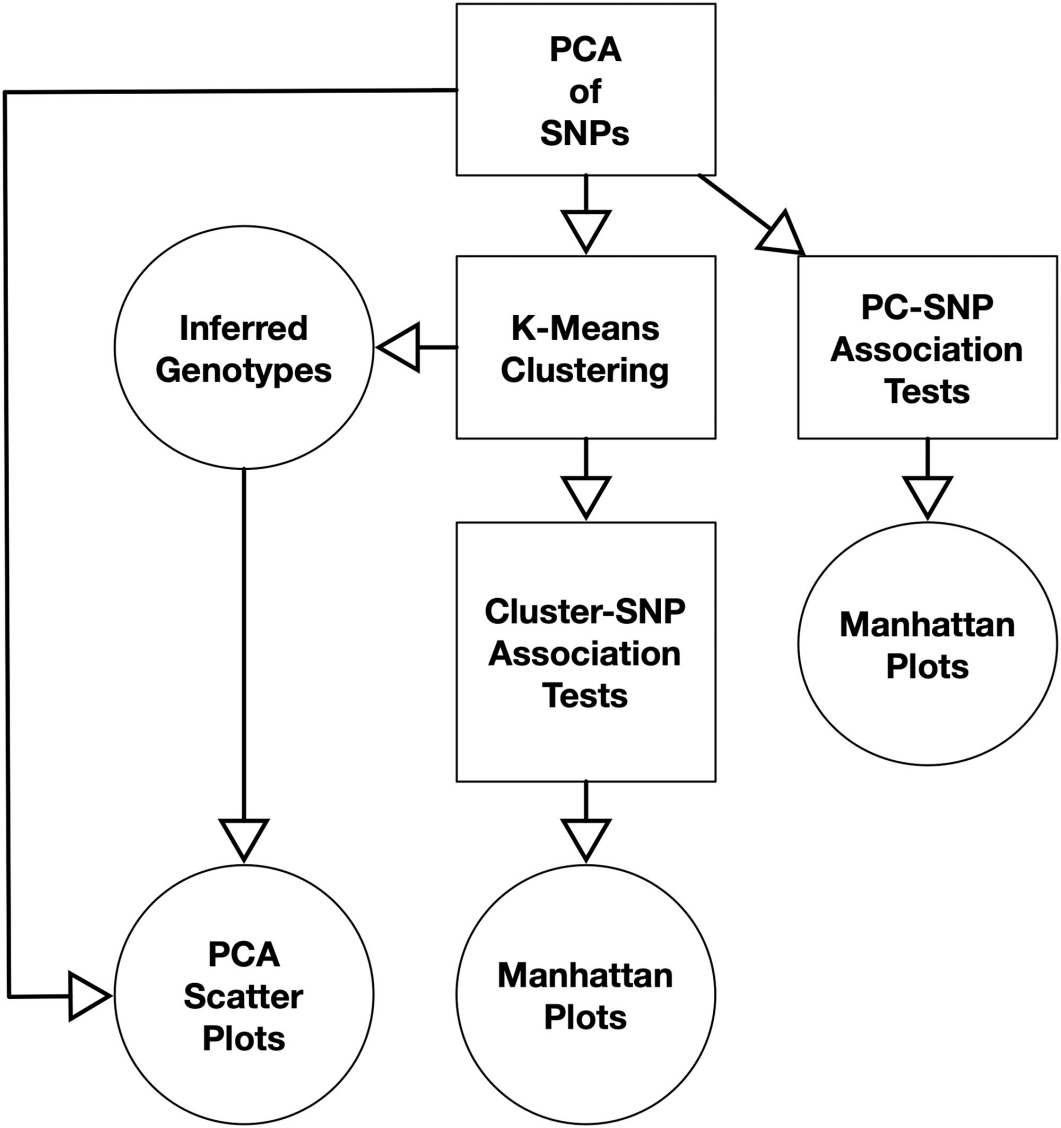
Workflows for detecting, localizing, and genotyping inversions. The three approaches (PCA with clustering, PC-SNP association testing, and Cluster-SNP association testing) all begin with performing PCA on a feature matrix generated from SNP data. K-Means clustering is performed using the PC coordinates to infer genotypes. The inferred genotypes and PC coordinates of the samples are represented using scatter plots. Association testing can be performed between the samples’ SNP genotypes and either the PC coordinates or cluster labels. The *p*-values from the association tests are plotted along the chromosome in a Manhattan plot to visualize the spatial distribution of the associations and detect and localize inversions.

An overview of the relationships of the methods is presented in Fig 1. All workflows began with PCA of SNP data encoded as a matrix. The PC coordinates of the samples can be visualized using a scatter plot; visualization identification of clusters can be interpreted to indicate population structure or inversions. The genotypes of the samples for each SNP can be tested for association with the samples’ coordinates along a PC, and the resulting −log_10_ of the *p*-values of the SNPs can be plotted along the chromosome arm for each PC in a Manhattan plot. A step-function like pattern in the Manhattan plots indicates that the PC captures an inversion and provides its location. Samples can be clustered (e.g., using k-means) by their PC coordinates to infer genotypes. Lastly, the genotypes of the samples for each SNP can be tested for association with the samples’ cluster membership and plotted in a Manhattan plot to determine if a given clustering captures an inversion and the inversion’s location.

We used implementations of the methods available in Asaph, our open-source toolkit for variant analysis available at https://github.com/rnowling/asaph under the Apache Public License v2. Asaph is implemented in Python using the Scikit Learn [42], Matplotlib [43], and Numpy / Scipy [44] libraries.

For each data set, we performed the following steps. First, we performed PCA. SNPs for each chromosome arm were imported into separate Asaph projects (import --workdir <workdir> --vcfgz <vcf.gz file> --compress with the default setting of categorical feature encoding). PCA was performed with 10 PCs (pca --workdir <workdir> train --n-components 10) with the default setting of a PCA model). Explained variances were output for each PC (pca --workdir <workdir> explained-variance-analysis); the number of PCs used in downstream analyses was chosen by looking for an “elbow” in the plots of the explained variances.

We clustered every data set six times (*k* = 1 to 6) and chose the appropriate number of clusters (*k*) by looking for an “elbow” in the resulting plot of the inertia (sum of squared errors) (python pc_analysis.py --coordinates <coordinates_fl.tsv> sweep-clusters --n-clusters 1 2 3 4 5 6 --n-components 1 2 --plot-fl <cluster-inertia.png>). We re-clustered the samples using the chosen value of *k* and output the cluster assignments to a text file (python pc_analysis.py --coordinates <coordinates_fl.tsv> output-clusters --n-clusters <k> --n-components 1 2 --labels-fl <cluster_labels.tsv>). Coordinates along the first 4 PCs were output from Asaph (pca --workdir <workdir> output-coordinates --selected-components 1 2 3 4 --output-fl <coordinates_fl.tsv>). PCA scatter plots were generated from the samples’ PC coordinates with samples colored by cluster (python pc_analysis.py --coordinates <coordinates_fl.tsv> plot-projections --pairs 1 2 3 4 --plot-dir <plot-dir> --labels-fl cluster_labels.tsv).

Secondly, we calculated PC-SNP associations (pca --workdir <workdir> snp-association-tests --components 1 2 3 4 --model-type logistic). The resulting *p*-values output to text files. One Manhattan plot was created per PC using the manhattan_plot.py script.

Lastly, cluster-SNP association tests were performed using the cluster labels (snp_association_tests --workdir <workdir> --populations cluster_labels.tsv) with the default settings (using the class probabilities to calculate the intercept, adjusting the training set through re-sampling, and the population labels as the dependent variable). A Manhattan plot was generated using the manhattan_plot.py script.

### Evaluation of inversion detection task

For each data set, we retrieved the inversions that had been detected in the original papers describing the data sets [17, 37–39]. For the clustering method, we recorded the number of clusters identified as optimal using the “elbow” in the inertia plots. If no confounding factors were present, we expected one cluster per inversion genotype present in the data set. For the PC-SNP association tests, we looked for a step-like function in the resulting Manhattan plots indicating an inversion; we did not require that an inversion was associated with a specific PC to count as detected. Lastly, for the Cluster-SNP association tests, we also looked for a step-like function in the resulting Manhattan plot.

### Evaluation of inversion genotype inference task

We retrieved the inversion genotypes for each sample in each data set from the original papers describing the data sets [17, 37–39]. We evaluated the agreement of the clusters with the known inversion genotypes. Clustering algorithms do not consistently return the same cluster IDs across runs, so we used a logistic regression model to test association between the cluster IDs (as a one-hot encoded categorical variable) and inversion genotypes. The model’s predictions were evaluated using a balanced accuracy metric to overcome class imbalance (not all genotypes were present in equal proportions) and weight each genotype equally.

### Evaluation of inversion localization task

We retrieved the coordinates for the inversion regions from the original papers describing the data sets [17, 37–39]. We estimated the observed inversion regions from the Manhattan plots generated from the PC-SNP and Cluster-SNP association tests. We compared the observed and expected regions qualitatively for agreement.

### Characterizations of inversions on the *Anopheles* 2R chromosome arms

We retrieved the 2R chromosome arm VCF file and sample IDs from the phase 1 AR3 data release from the 1000 *Anopheles* Genome [17] project web site. We used VCFtools to select the 150 Burkina Faso *Anopheles gambiae* and *Anopheles coluzzii* samples, biallelic SNPs, and generate three VCF files (both species, only *An. gambiae*, and only *An. coluzzii*). We followed the workflow described above to generate PCA scatter plots and Manhattan plots from cluster-SNP and PC-SNP association tests.

## Results

We formed three test sets from publicly-available insect population genomics data sets (see below and Table 2). We used these test data to evaluate three methods (PCA with clustering, cluster-SNP association tests, and PC-SNP association tests) across three problems (inversion detection, inversion genotype inference, and inversion localization). Lastly, we applied this framework to SNPs from 150 Burkina Faso *Anopheles* samples from the 1000 *Anopheles* Genomes project [17] and found inversions (2Rc and 2Rd) that were not previously annotated.

**Table 2:** Characterization of SNP data sets. A benchmark data set for evaluating methods for inversion detection using using SNP data was formed from data for three insect species (*D. melanogaster* [37, 38], *An. gambiae* and *coluzzii* [17, 39]). The chromosome arms were organized into three test cases (negatives, positive drawn from a single population, and positive drawn from multiple populations) based on known inversion genotypes from previous papers. We analyzed SNPs from the 2R chromosome arms of *An. gambiae* and *coluzzii* but do not include these data in our benchmark data set since not all inversions were fully characterized. For each chromosome arm, the geographic locations in which the samples were collected, species of the samples, number of samples, inversions identified in these data by the original authors and their frequencies, and the number of SNPs are provideded.

Test Case	Data Source	Location	Species	Chrom.	Samples	Inversions (Frequency)	SNPs
Negative	[37, 38]		*D. mel*.	3L	192*		896,257
Negative	[39]	BCMT	*An. gam*. and *col*.	3L	34		1,329,375
Negative	[17]	B	*An. gam*. and *col*.	3L	150		7,449,486
Single	[37, 38]		*D. mel*.	2L	198	*In(2L)t* (14.4%)	910,880
Single	[37, 38]		*D. mel*.	2R	198	*In(2R)NS* (12.1%)	740,948
Single	[37, 38]		*D. mel*.	3R	198	*In(3R)Mo* (18.7%), *In(3R)p* (7.1%), *In(3R)k* (8.1%)	884,009
Multiple	[17]	B	*An. gam*. and *col*.	2L	150	2La (94.7%)	8,296,600
Multiple	[17]	B	*An. gam*.	2L	81	2La (90.7%)	
Multiple	[39]	BCMT	*An. gam*. and *col*.	2L	34	2La (54.4%)	
Other	[17]	B	*An. gam*. and *col*.	2R	150	2Rb (59.3%)	11,332,702
Other	[17]	B	*An. gam*.	2R	81	2Rb (82.1%)	11,332,702
Other	[17]	B	*An. col*.	2R	69	2Rb (31.1%)	11,332,702
Other	[17]	B	*An. col*.	2L	69	2La (99.3%)	8,296,600

* Inversions were present in only six samples, which we removed; B: Burkina Faso, C: Cameroon, M: Mali, and T: Tanzania

### Formation of three test sets

We constructed three test sets (negative for inversions, inversions in samples from a single species and population, and inversions in samples from multiple species and/or populations) from previously-published data [17, 37–39] (see Table 2).

**Fig 2:**
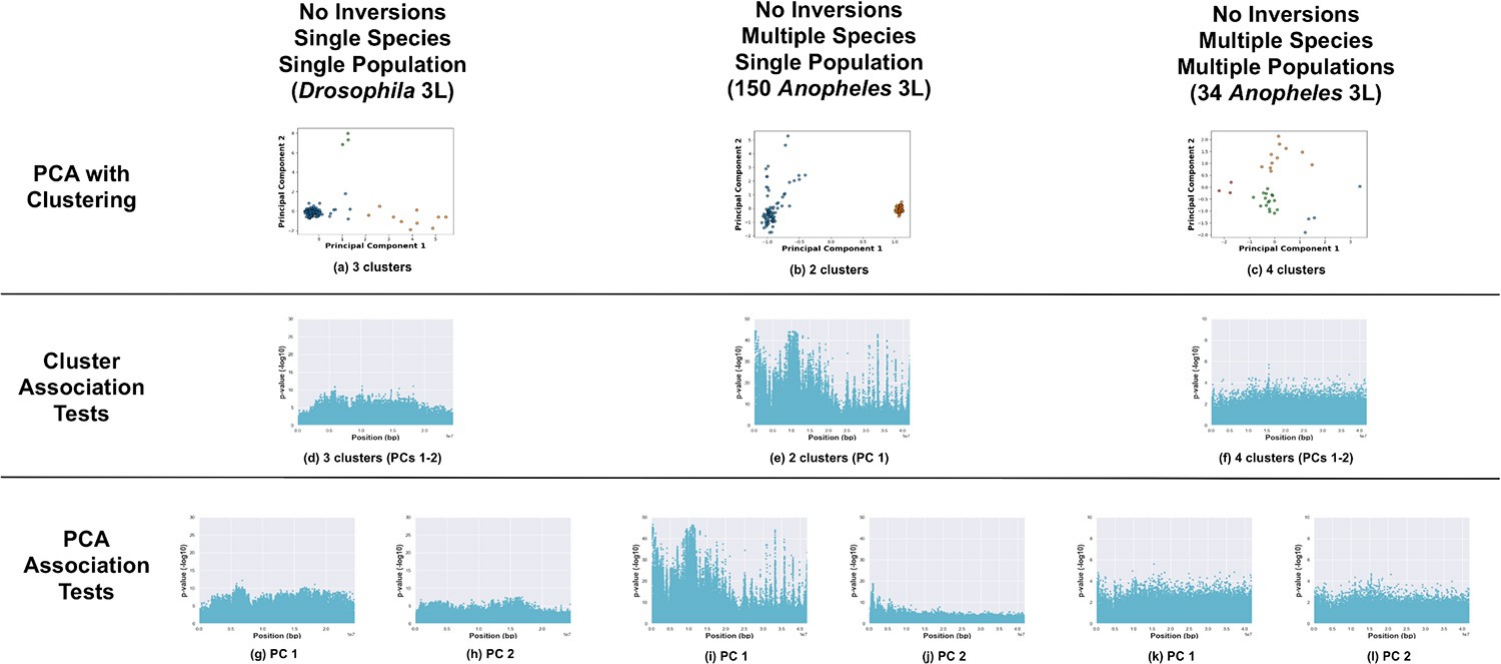
Negative cases. Analysis of chromosome arms without known major inversions (*Drosophila* 3L—6 samples with inversion excluded (see Methods), 150 *Anopheles* 3L, and 34 *Anopheles* 3L). (a—c) PCA of samples, clustered with k-means, and colored by cluster. Manhattan plots visualizing *p*-values from association tests against sample cluster IDs (d—f) and PC coordinates (g—l, one Manhattan plot per PC).

Negative test case: We selected the *Drosophila melanogaster* 3L, 150 Burkina Faso *Anopheles* 3L, and 34 *Anopheles* 3L chromosome arms (see Fig 2). The *Anopheles* 3L chromosome arms have no known high or moderately high frequency inversions. The *Drosophila* 3L chromosome arm had several low-frequency inversions (*In(3L)M*, *In(3L)K*, and *In(3L)P*), so we removed the six samples with heterozygous or homozygous inverted genotypes. The *Drosophila* samples were drawn from a single population, the 150 *Anopheles* samples included two species (*An. gambiae* and *coluzzii*) from the same geographical location (Burkina Faso), and the 34 *Anopheles* samples included two species (*An. gambiae* and *coluzzii*) from four geographic locations (Burkina Faso, Cameroon, Mali, and Tanzania).

Two of the test sets were positive for inversions. The first test set (single positive) was formed from three *Drosophila* chromosome arms (2L, 2R, and 3R). The samples were all drawn from the same population and species to avoid these confounding factors. The 2L and 2R chromosome arms each contained a single prominent inversion (*In(2L)t*, *In(2R)NS*) each with all three inversion genotypes present. Three separate inversions (*In(3R)P*, *In(3R)K*, and *In(3R)Mo*) were present on the 3R chromosome arm; the homozygous inverted and heterozygous genotypes of the inversions are mutually exclusive with each other, which complicates the analysis (see S1 File).

The last test set (multiple positive) included data from multiple species and/or from multiple geographic locations (150 Burkina Faso *Anopheles* 2L, 81 Burkina Faso *An. gambiae* 2L, and 34 *Anopheles* (4 locations) 2L). All samples had been previously karyotyped for the 2La inversion [17, 39]. Detection of the 2La inversion in the 150 Burkina Faso samples was complicated since not all inversion genotypes are present in both species; none of the samples had the homozygous standard genotype and only a single *An. coluzzii* sample is heterozygous (see Table 4).

In the 16 *Anopheles* genomes samples, the 2La inversion genotypes were associated with both species and locations. Samples from Cameroon were primarily homozygous for the inverted orientation, while samples from Burkina Faso and Mali were primarily homozygous for the standard orientation (see Table 3). Five samples from across locations are heterozygous. All three genotypes were observed in *An. gambiae* samples, while *An. coluzzii* samples were homozygous for either the standard or inverted orientations (see Table 4).

**Table 3:** Occurrences of 2La inversion genotypes by location for 34 *Anopheles* samples. The 2La inversion genotypes for the 34 *An. gambiae* and *coluzzii* samples from [39] by were analyzed for association with geographic location. The homozygous inverted genotype was observed primarily in samples from Cameroon, while the homozygous standard genotype was observed in samples only from Burkina Faso and Mali. Association of the inversion genotypes with geographic location prevents correction for potential confounding effects for this data set.

Location	Homo. Std.	Hetero.	Homo. Inv
**Burkina Faso**	5	2	0
**Cameroon**	0	1	15
**Mali**	8	0	0
**Tanzania**	0	2	1

**Table 4:** Occurrences of 2La inversion genotypes by *Anopheles* species and data set. The 2La inversion genotypes for the 34 *An. gambiae* and *coluzzii* samples from [39] and 150 *An. gambiae* and *coluzzii* samples from [17] were analyzed for association with species. The two papers do not agree on the definitions of the standard and inverted orientations. The homozygous standard inversion genotype was not observed in the 150 Burkina Faso samples but was dominant in the Burkina Faso samples from [39] (see Table 3). Likewise, the homozygous inverted genotype was not observed in the Burkina Faso samples from [39] but was dominant among the 150 Burkina Faso samples.

Data Source	Species	Homo. Std.	Hetero.	Homo. Inv
[17]	*An. coluzzii*	0	1	68
[17]	*An. gambiae*	0	15	66
[39]	*An. coluzzii*	3	0	8
[39]	*An. gambiae*	10	5	8

The 2La genotype labels may not be consistent between the 16 *Anopheles* genomes and 1000 *Anopheles* genomes data. The 2La homozygous inverted genotype was not observed among the 7 Burkina Faso samples from the 34 total *Anopheles* samples, while the 2La homozygous standard orientation was not observed among the 150 Burkina Faso *Anopheles* samples (see Table 4). We suspect that the data sets disagree on which orientations are standard and inverted.

### Evaluation on inversion detection task

**Fig 3:**
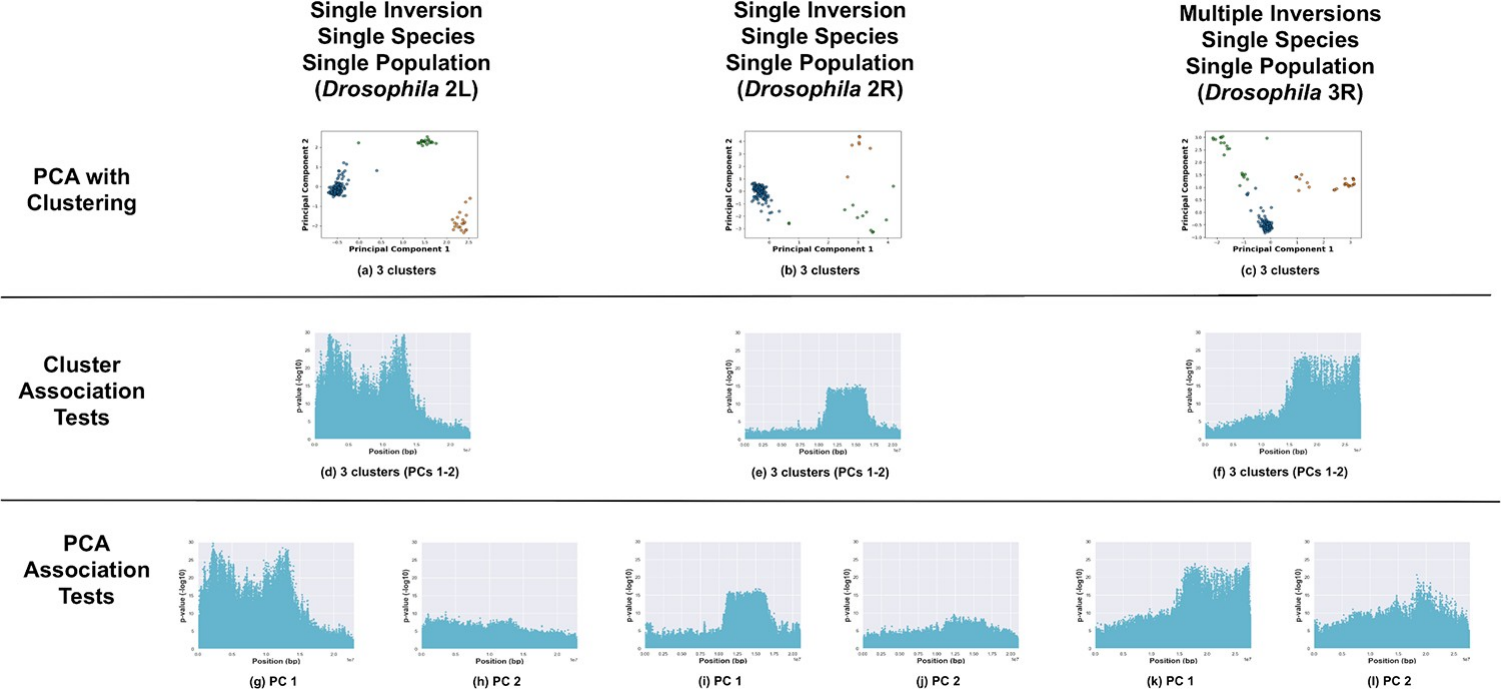
Positive cases with a single species. Analysis of chromosome arms with known major inversions in samples drawn from a single species (*Drosophila* 2L, 2R, and 3R). (a—c) PCA of samples, clustered with k-means, and colored by cluster. Manhattan plots visualizing *p*-values from association tests against sample cluster IDs (d—f) and PC coordinates (g—l, one Manhattan plot per PC).

**Fig 4:**
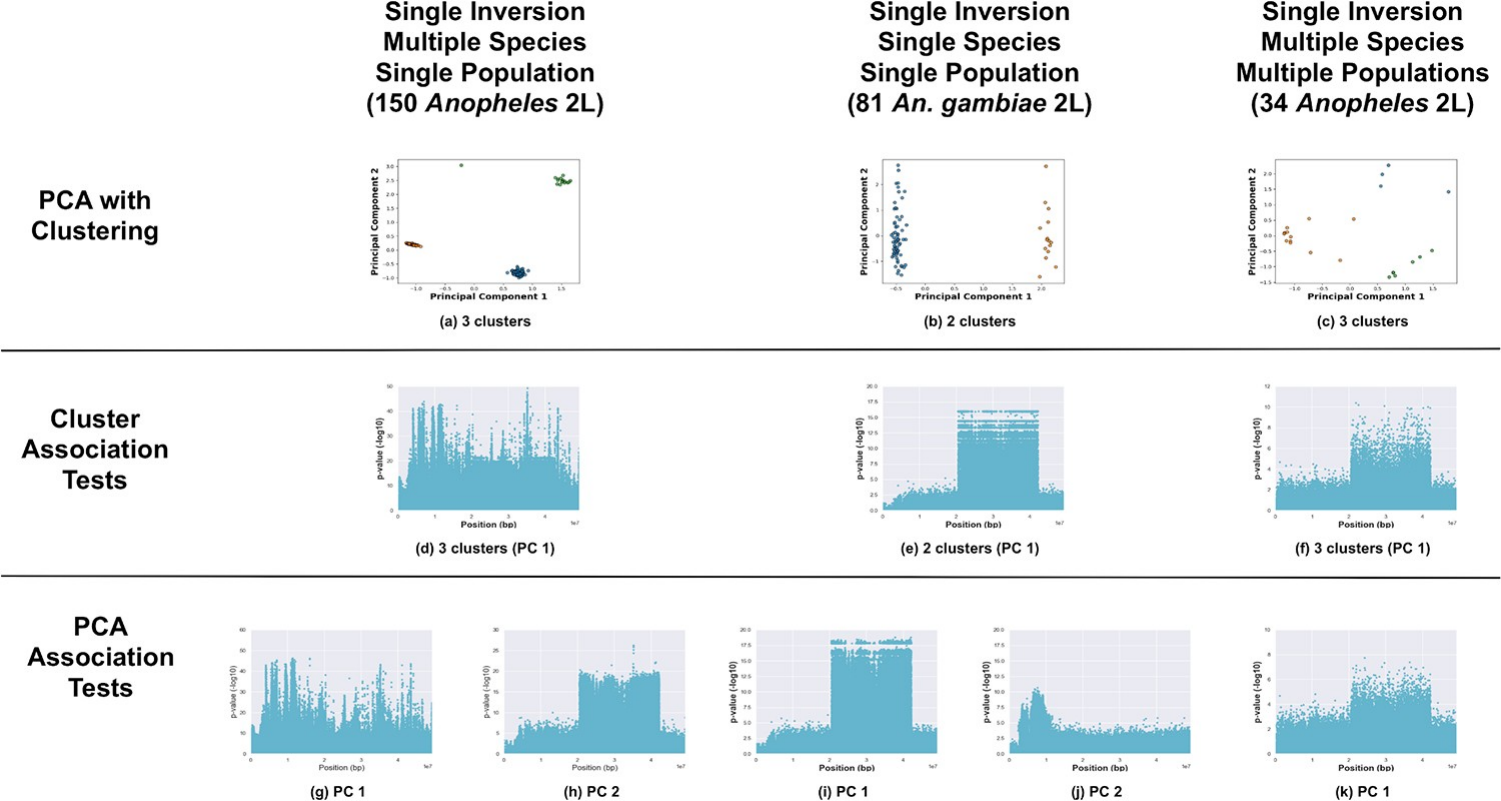
Positive cases with a multiple species and/or populations. Analysis of the 2L *Anopheles* chromosome arm with known major inversions in samples drawn from multiple species and/or locations (150 *Anopheles* from Burkina Faso, 81 *Anopheles gambiae* samples of the 150 *Anopheles* samples, and 34 *Anopheles gambiae* and *coluzzii* samples from four geographic locations). (a—c) PCA of samples, clustered with k-means, and colored by cluster. Manhattan plots visualizing *p*-values from association tests against sample cluster IDs (d—f) and PC coordinates (g—k, one Manhattan plot per PC).

All three methods (PCA with clustering, cluster-SNP association tests, and PC-SNP association tests) are capable of detecting inversions. Here we illustrate results for: no inversions present with single or multiple species and/or populations (Fig 2); inversions present with a single species and a single population (Fig 3); and inversions present with multiple species and/or populations (Fig 4).

An inversion (without confounding factors) is expected to segregate samples into two or three clusters (one per inversion genotype present in the data) in PCA. We first evaluated test cases with no inversions (see Fig 2a–2c). One large cluster and some outliers were observed in the PCA for *Drosophila* 3L; k-means identified three clusters as optimal for fitting the data (see S1 File). We analyzed the *Anopheles* 3L chromosome arm using both *Anopheles* data sets. The 150 Burkina Faso samples segregated into two clusters (corresponding to the two species) in the PCA, while the 34 samples formed four clusters corresponding to the four geographic areas. The two-cluster patterns observed for 3L and 2L (with the single 2La inversion) for the 150 Burkina Faso samples were similar (compare Figs 2b and 4b) despite different causes (two species versus the 2La inversion); the clusters present a second example that could be misinterpreted without prior knowledge of the inversion status of the samples.

Secondly, we analyzed the test cases of inversions present with a single species and population (see Fig 3a–3c). Three clusters were present for the two arms (*Drosophila* 2L and 2R) each with a single inversion (as expected). For the case with multiple mutually-exclusive inversions (*Drosophila* 3R), more than three clusters would be expected due to combinations of different inversion genotypes. K-means identified three clusters, however, as the optimal fit according to the “elbow” in the inertia plot (see S1 File). Without prior knowledge of the inversions, the three clusters could be misinterpreted as indicating the presence of a single inversion.

Lastly, we evaluated the third set of test cases of inversions present in samples from multiple species and/or multiple populations (see Fig 4a–4c). We focus on the 2La inversion in the two *Anopheles* data sets. When both species were analyzed together, the samples from both data sets segregated into three clusters in the PCA (see Fig 4a and 4c). The three clusters did not correspond to the three possible inversion genotypes, but to combinations of the inversion genotypes and species. (Not all inversion genotypes were present in the samples.) We isolated and separately analyzed the 81 *An. gambiae* samples from the 150 Burkina Faso samples. The *An. gambiae* samples segregated into two clusters (see Fig 4b), corresponding to the two inversion genotypes that were present.

Cluster-SNP association tests detected inversions more accurately than PCA and clustering alone. Inversions were indicated by a “step” function in the resulting Manhattan plots. For the test cases of inversions present in a single species and population, the cluster-SNP association tests were consistent with PCA. The *Drosophila In(2L)t and In(2R)NS* inversions were readily identified in the Manhattan plots (see Fig 3d and 3e), while the method was unable to differentiate between the multiple inversions on 3R (see Fig 3f). For the negative test cases, there were either few SNPs with strong associations or associated SNPs were distributed widely across the chromosome arms with no clear contiguous step-function pattern indicative of an inversion (see Fig 2d–2f); the method successfully avoided false positives even when inversion-like cluster patterns (e.g., *Anopheles* 3L) were present in the PCA.

Inversion detection with multiple species proved more challenging with the cluster-SNP association tests (see Fig 4d–4f). The cluster-SNP association tests failed to identify 2La in the 150 Burkina Faso samples (see Fig 4d). The 2La inversion was clearly indicated in the analysis of the subset of 81 *An. gambiae* samples (see Fig 4e). The presence of both species was not problematic in the analysis of the 34 *Anopheles* samples data set; 2La was clearly visible (see Fig 4f).

The PC-SNP association tests were both accurate and easy to apply. The PC-SNP association tests are performed for each PC and do not depend on identifying representative clusters. The *Drosophila In(2L)t and In(2R)NS* inversions were readily identified in the Manhattan plots of associations against PC 1 for each arm; as with the other methods, the multiple inversions on 3R were misrepresented as a single inversion (see Fig 3g–3l). Like the cluster-SNP association tests, the negative test cases either had few strongly-associated SNPs or associated SNPs were distributed widely throughout the chromosome arms with no apparent step-function pattern (see Fig 2g–2l).

The PC-SNP association tests method was most successful at identifying inversions with multiple species and/or populations (see Fig 4g–4l). For the 150 Burkina Faso samples, the 2La inversion was detected in the Manhattan plot for associations against PC 2 and with even greater clarity in associations with PC 1 for the 81 *An. gambiae* samples. Lastly, when applied to the 34 *Anopheles* samples from four locations, 2La was visible in the association tests with PC 1.

### Evaluation on inversion genotype inference task

Of the three methods, only PCA-clustering was able to infer samples’ inversion genotypes. We evaluated the agreement of the inferred inversion genotypes with the experimentally-determined inversion genotype labels for our data set (see Table 5). Cluster assignments (labels) were not always ordered consistently (e.g., randomly ordered). We trained logistic regression models to predict the samples’ genotypes from the cluster labels and evaluated the predictions using the balanced accuracy metric. This metric avoids erroneously high accuracy scores when samples in a small class are mislabeled.

**Table 5:** Genotype inference task. We evaluated a single methods (PCA with clustering) on the genotype inference task (which inversion genotype does a sample have?) using two benchmark test cases (positive from a single population and positive from multiple populations). Note that the two association-testing methods are not able to infer genotypes. For each chromosome arm used, we indicated known inversions, how many genotypes are present in the data set, and a measure of balanced accuracy calculated from the cluster predictions. The *D. melanogaster* 3R chromosome arm has three mutually-exclusive inversions, which we list separately.

Test Case	Chrom.	Inversion	Present Genotypes	Clusters	Balanced Accuracy
Single	*D. mel*. 2L	*In(2L)t*	3	3	93.3%
Single	*D. mel*. 2R	*In(2R)NS*	3	3	94.4%
Single	*D. mel*. 3R	*In(3R)Mo*		3	60.7%
Single		*In(3R)p*		3	43.3%
Single		*In(3R)K*		3	55.0%
Multiple	150 *An. gam*. and *col*. 2L	2La	2	3	66.7%
Multiple	81 *An. gam*. 2L	2La	2	2	100.0%
Multiple	34 *An. gam*. and *col*. 2L	2La	3	4	100.0%

We evaluated clustering in terms of accuracy of inferring inversion genotypes. Inversion genotypes were retrieved from the original papers describing the data [17, 37–39]. Association of the known genotypes with the cluster labels was measured using balanced accuracy. *Could not resolve multiple, mutually-exclusive inversions

Inversion genotype inference was straight-forward and accurate for tests cases where a single inversion was present in samples from a single species and population (Fig 3). For the *Drosophila* 2L and 2R chromosome arms, the inversion genotypes predicted the cluster labels with accuracies of 93.3% (*In(2L)t*) and 94.4% (*In(2R)NS*). Genotype inference was less successful for the *Drosophila* 3R chromosome arm with multiple mutually-exclusive inversions (*In(3R)K*, *In(3R)mo*, and *In(3R)p*) (Fig 3). We evaluated different combinations parameters (PCs 1-2, k = 3-7). In the best case (k = 3, PCs 1 and 2), balanced accuracies for predicting cluster assignments from karyotype labels were 55.0% (*In(3R)K*), 60.7% (*In(3R)mo*), and 43.3% (*In(3R)p*).

We then evaluated the test cases with multiple species and/or populations (Fig 4). The 150 Burkina Faso samples segregated into three clusters (with one outlier point) in the PCA of the 2L SNPs. The clusters corresponded to combinations of the species and 2La inversion genotypes (only the heterozygous and homozygous inverted genotypes were present in the samples) and resulted in a balanced accuracy of 66.7%. Clustering of the 81 *An. gambiae* and 34 *Anopheles* samples predicted the 2La genotypes with 100% balanced accuracies.

### Evaluation on inversion localization task

Two of the methods (cluster- and PC-SNP association tests) were able to localize inversions. We qualitatively compared the step-function patterns in the Manhattan plots with reported genomic coordinates (see Table 6). The strongly-associated SNPs on 2L and 2R extended past the reported regions for the *Drosophila* *In(2L)t* and *In(2R)NS* inversions on both ends (see Fig 3). The strongly-associated SNPs spanned approximately 0—16 Mbp in the 2L Manhattan plots versus the reported region of 2.2—13.2 Mbp for *In(2L)t* [37]. Similarly, for 2R, strongly-associated SNPs spanned approximately 10—17.5 Mbp versus the reported region of 11.3—16.2 Mbp. The *In(3R)P*, *In(3R)K*, and *In(3R)Mo* inversions were reported to span 12.6—20.6 Mbp, 7.6 Mbp—22.0 Mbp, and 17.2—24.9 Mbp, respectively [37]. In the Manhattan plots, the inversion region on 3R spans from approximately 15 Mbp to the end of the chromosome arm and overlapped all three inversions.

**Table 6:** Inversion localization task. We evaluated the two association-testing methods (PC-SNP and Cluster-SNP association tests) on the inversion localization task (what region is spanned by an inversion?) using two benchmark test cases (positive from a single population and positive from multiple populations). Note that the two PCA scatter plot method is not able to localize inversions. For each chromosome arm used, we indicated known inversions, the expected ranges, and the ranged identified be each method. The *D. melanogaster* 3R chromosome arm has three mutually-exclusive inversions, which we list separately.

Test Case	Chrom.	Inversion	Exp. Range (Mb)	PC-SNP Obs. Range (Mb)	Cluster-SNP Obs. Range (Mb)
Single	*D. mel*. 2L	*In(2L)t*	2.2–13.2	start–16.0 (PC1)	start–16.0
Single	*D. mel*. 2R	*In(2R)NS*	11.3–16.2	10.0–17.5 (PC1)	10.0–18.0
Single	*D. mel*. 3R	*In(3R)Mo*	12.6–20.6	14.0–end* (PC1)	14.0–end*
Single		*In(3R)p*	7.6–22.0 Mb	14.0–end* (PC1)	14.0–end*
Single		*In(3R)K*	17.2–24.9 Mb	14.0–end* (PC1)	14.0–end*
Multiple	150 *An. gam*. and *col*. 2L	2La	20.0–45.0	20.0–43.0 (PC2)	start–end^†^
Multiple	81 *An. gam*. 2L	2La	20.0–45.0	20.0–43.0 (PC1)	20.0–43.0
Multiple	34 *An. gam*. and *col*. 2L	2La	20.0–45.0	20.0–43.0 (PC1)	20.0–43.0

We evaluated the PC-SNP and Cluster-SNP association test methods on localizing inversions. We compared the range of inversions observed in the Manhattan plots created from these two methods with the coordinates described for these inversions in prior work [37–39, 45, 46].

*Could not resolve multiple, mutually-exclusive inversions

^†^Could not resolve 2La

The association test methods localized the 2La inversion more accurately and consistently than the *Drosophila* inversions. The *Anopheles* 2La inversion spans approximately 20.0—45.0 Mbp on 2L [39, 45, 46]. Where visible in the Manhattan plots for the cluster- and PC-SNP association tests, SNPs associated with 2La inversion were consistently localized to the 20.0—43.0 Mbp region (see Fig 4).

### Characterizations of inversions on the *Anopheles* 2R chromosome arm

**Fig 5:**
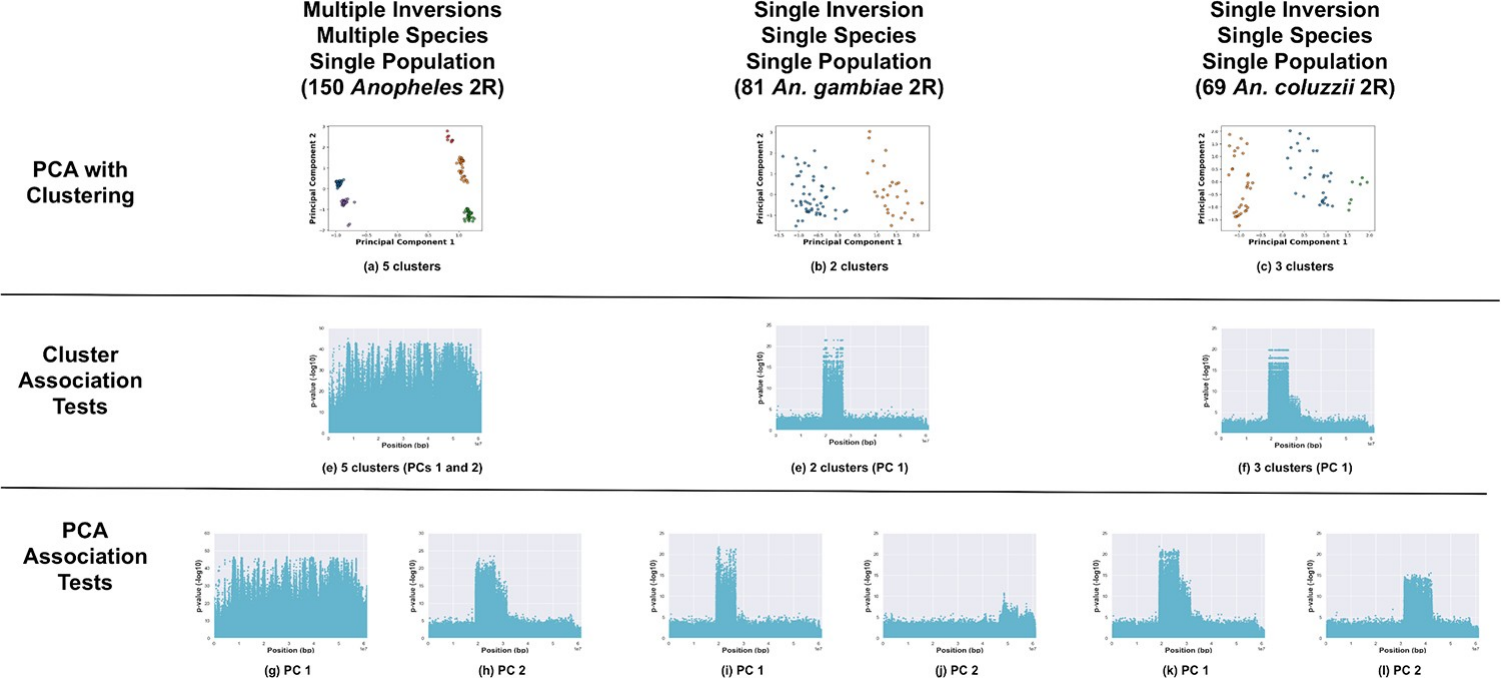
*Anopheles* 2R chromosome arm. Analysis of the 2R chromosome arm of the 150 *Anopheles* samples from Burkina Faso (all samples, 81 *Anopheles gambiae* samples, and 69 *Anopheles coluzzii* samples). (a—c) PCA of samples, clustered with k-means, and colored by cluster. Manhattan plots visualizing *p*-values from association tests against sample cluster IDs (d—f) and PC coordinates (g—k, one Manhattan plot per PC).

We applied the inversion detection methods to the 2R chromosome arm in the 150 Burkina Faso *Anopheles* samples from the 1000 *Anopheles* Genome project (see Fig 5). The 1000 *Anopheles* Genome project samples were karyotyped for the 2Rb inversion but karyotype labels for other previously-known 2R inversions had not been made available to our knowledge [17].

Five clusters were present in PCA of the SNPs (see Fig 5a), but no inversions were visible in the cluster-SNP association tests (despite expecting the 2Rb inversion to be visible). The PC-SNP association tests revealed the 2Rb inversion (and possibly, the 2Rc inversion) (see Fig 5g and 5h).

The analysis was confounded by analyzing the species together, so we divided the samples by species and analyzed each species separately. We observed the pattern for 2Rb in *An. gambiae* as expected (see Fig 5b, 5e, 5i and 5j). No other 2R inversions appeared to be present in the *An. gambiae* samples.

We observed multiple 2R inversions (2Rb, 2Rc, and 2Rd) in the *An. coluzzii* samples. The 2Rc inversion is adjacent to 2Rb and when the two inversions appear together, they are designated as the 2Rbc system [45, 46]. The 2Rbc inversion genotype was visible in both the cluster-SNP associations and PC-SNP associations for PC 1 (see Fig 5f and 5k). The presence of 2Rc (2Rbc) in some of the *An. coluzzii* samples explains why the karyotypes from the two species did not cluster together along PC 2 when the 150 samples were analyzed together. The 2Rd inversion was observed in the PC-SNP associations for PC 2 see Fig 5l).

## Discussion

While experimental techniques such as Fluorescence *in situ* hybridization (FISH) are the most accurate way to identify inversions [47–49], the chromosomes of many non-model insect species are not visible under a microscope, and we must turn to computational methods. In human genomics, the most popular methods for detecting structural variations such as inversions are based on alignment of reads to a reference genome. Inversion breakpoints can be discovered by checking for cases where either paired-end sequence data align unexpectedly (e.g., [50–53]). Breakpoints in *Anopheles* mosquitoes are characterized by long repeated sequences [47, 48] which have prohibited breakpoint detection with alignment-based methods [54, 55]. Methods for detecting inversions from SNP data (e.g., see Table 7) are a promising alternative for analyzing inversions in non-model organisms (e.g., [16, 18, 25–27, 39, 57].

**Table 7:** Summaries of inversion analysis tools. Details of existing software tools that were either designed or can be applied to inversion analysis using SNP data are summarized.

	SNPRelate	PCAdapt	Asaph	inveRsion	invClust	EIGENSOFT	PLINK
**Paper**	[34]	[35, 36]	[16]	[56, 62]	[15]	[29, 31]	[63, 64]
**Language**	R	C and R	Python	R	R	C	C
**Summary**	SNPRelate provides parallel implementations of PCA for SNP data and the ability to perform correlation testing between PC coordinates and SNP genotypes. Although not designed for inversion detection, SNPRelate can be applied to inversion detection using PCA scatter and Manhattan plots.	PCAdapt uses PCA to infer population structure and assumes variants with strong associations with the PC coordinates are under local selection. Although not designed for inversion detection, PCA scatter plots and variant p-values from association tests can be used to detect inversions with scatter and Manhattan plots, respectively.	Asaph uses PCA, clustering, and association tests to detect, genotype, and localize inversions.	inveRsion identifies changes in linkage disequilibrium along the chromosome arm from SNP data to find inversion breakpoints.	Developed by the authors of inveRsion, invClust performs PCA and clustering of samples with Gaussian mixture models to perform inversion genotype inference. Inversions can first be detected and localized by inveRsion and then invClust can be applied to SNPs in the inversion region.	EIGENSOFT provides analysis of population structure using PCA.	PLINK can perform population inference with PCA and perform regression with quantitative traits. Although not the intended purpose, these techniques can be used for inversion analysis.
**Year Released**	2012	2014 (C) / 2016 (R)	2018	2012	2015	2006	2007
**Inversion Detection**	Yes	Yes	Yes	Yes	Yes	Yes	Yes
**Genotyping**	Yes	Yes	Yes	No	Yes	Yes	Yes
**Localization**	Yes	Yes	Yes	Yes	No	No	Yes
**Software Link**	https://www.bioconductor.org/packages/release/bioc/html/SNPRelate.html	https://cran.r-project.org/web/packages/pcadapt/index.html	https://github.com/rnowling/asaph	https://www.bioconductor.org/packages/release/bioc/html/inveRsion.html	https://rdrr.io/github/isglobal-brge/invClust/	https://github.com/DReichLab/EIG	https://www.cog-genomics.org/plink/1.9/

We generated a new benchmark for evaluating computational inversion analysis by gathering and curating publicly-available SNP data sets from the *Drosophila* Genetic Reference Panel v2 (DGRP2) [37, 38], 1000 *Anopheles* Genomes project [17], and 16 *Anopheles* Genomes project [39]. *Drosophila melanogaster* and *Anopheles* species have large polytene chromosomes that can be seen directly under a microscope [47–49]; consequently, data from these species are well-suited to evaluating inversion detection methods. Samples in these data sets were genotyped for several well-studied large inversions (by the original researchers). These data provided interesting test cases such as complex relationships between inversions genotypes and population structure (the *Anopheles* samples) and each other (e.g., inversions of the 3R chromosome arm of the *D. melanogaster* samples). This data set will be useful in future work on inversion detection methods. We provided scripts and metadata in our public repository for Asaph so that others can easily regenerated the benchmark data set from the original data.

We previously described a family of PCA-based inversion analysis methods for SNP data [16]. These methods are implemented in and distributed through our open-source software package Asaph (https://github.com/rnowling/asaph). In our original work, we only validated the framework on the 34 *An. gambiae* and *coluzzii* samples from the 16 *Anopheles* Genomes project [39]. Here, we performed a more in-depth validation using the new benchmark. We looked at three tasks: inversion detection, inference of inversion genotypes, and inversion localization. Analyzing samples from multiple species or locations produced an inversion-like cluster pattern even when no inversions were present. For example, the combination of two species and the presence of only two out of three 2La genotypes in 150 Burkina Faso *Anopheles* samples resulted in three clusters that could be misinterpreted as inversions alone. Our results are expected given the wide range of use cases for PCA beyond inversion detection such as analyzing population structure [29, 31]. Ideally, we would only analyze samples from a single species and geographic location; unfortunately, this might not always possible.

Cluster- and PC-SNP association tests were substantially easier to interpret than the PCA-clustering approach. All inversions were detected by the PC-SNP association test method, even in cases with confounding factors (e.g., *Anopheles* 2La inversion) (see Table 8). The cluster-SNP association test methods identified most of the inversions except in the case of jointly analyzing the 150 Burkina Faso *An. gambiae* and *coluzzi* samples. Neither method was able to deconvolve the the multiple mutually-exclusive inversions on the *Drosophila* 3R chromosome arm, although both methods did detect the presence of inversions in that region.

**Table 8:** Inversion detection task. We evaluated three methods (PCA with clustering, PC-SNP association testing, and Cluster-SNP association testing) on the inversion detection task (is an inversion present?) using our three benchmark test cases (negative, positive from a single population, and positive from multiple populations). For each chromosome arm used, we indicated known inversions and whether the inversion was detected by a given method. The *D. melanogaster* 3R chromosome arm has three mutually-exclusive inversions, which we list separately.

Test Case	Chrom.	Inversion	Clusters	PC-SNP	Cluster-SNP
Negative	*D. mel*. 3L	None	1	No	No
Negative	34 *An gam*. and *col*. 3L	None	4	No	No
Negative	150 *An gam*. and *col*. 3L	None	2	No	No
Single	*D. mel*. 2L	*In(2L)t*	3	Yes (PC 1)	Yes
Single	*D. mel*. 2R	*In(2R)NS*	3	Yes (PC 1)	Yes
Single	*D. mel*. 3R	*In(3R)Mo*	3	Yes (PC 1)*	Yes*
Single		*In(3R)p*	3	Yes (PC 1)*	Yes*
Single		*In(3R)K*	3	Yes (PC 1)*	Yes*
Multiple	150 *An. gam*. and *col*. 2L	2La	3	Yes (PC 2)	No
Multiple	81 *An. gam*. 2L	2La	2	Yes (PC 1)	Yes
Multiple	34 *An. gam*. and *col*. 2L	2La	4	Yes (PC 1)	Yes

We compared inversions detected by the three methods to the known inversion karyotypes for these data sets taken from the original papers describing the data [17, 37–39]. If an inversion was present with no population structure, three clusters corresponding to three possible genotypes (which may not all be present) would be expected.

*Multiple, mutually-exclusive inversions were detected as a single inversion by our methods.

Of the three approaches, only the PCA-clustering method was capable of inferring inversion genotypes. Clustering accurately inferred inversion genotypes for the *Drosophila* *In(2L)t* and *In(2R)NS* and *Anopheles* 2La inversions but not the mutually-exclusive *Drosophila* *In(3R)K*, *In(3R)mo*, and *In(3R)p* inversions. These results were consistent with difficulties deconvolving the 3R inversions on the detection and localization tasks.

Two of the methods (cluster- and PC-SNP association tests) were able to localize large inversions. Breakpoints for large inversions in insects can occur in areas with long runs of simple tandem repeats [47, 48], which inhibit accurate and consist determination of genomic coordinates. In the *Drosophila* data sets, strongly-associated SNPs extended past the previously-recorded genomic coordinates for *In(2L)t* and *In(2R)NS* by ≈2 Mbp on each side. The 3R inversions were ambiguous and difficult to detect due to mutual exclusion and overlaps but the locations of the strongly-associated SNPs best fit the *In(3R)Mo* inversion. In contrast, the 2La inversion was localized accurately and consistently in the two *Anopheles* data sets. Repressed recombination extending outward several megabases from inversion break points beyond has been observed in *Drosophila* [58]; it is interesting that the methods detect this reduced recombination.

Both the clustering (for detection and genotype inference) and cluster-SNP association test methods required careful selection of the PCs and number of clusters. Incorrect choices lead to inaccurate inversion detection, genotype inference, and inversion localization. With no parameters to tune, the PC-SNP association test method was both the easiest to use and most reliable of the three methods for inversion detection and localization. We found it useful to first detect and localize inversions with the PC-SNP association test method and then guide the selection of the PCs and number of clusters by reproducing the results with the cluster-SNP association test method. Only once the appropriate PCs and number clusters were identified did we attempt genotype inference. Association testing enabled more accurate inversion detection, validation of the clustering parameters, and localization of inversions. To test this further, we applied the three approaches to analyze SNPs from the 2R chromosome arm of the 150 Burkina Faso *Anopheles* samples. At the time of our analysis, karyotype labels were only publicly-available for the 2Rb inversion [17] but not other known 2R inversions [11, 45, 46]. We identified the potential presence of the 2Rc and 2Rd inversions in the *An. coluzzii* samples. During the revision stage for this paper, experimental work identifying the presence of the 2Rc and 2Rd inversions in these particular *An. coluzzii* samples became available [59, 60]. The experimental work both validated our results and confirmed the utility of the association-test methods discussed here.

## Conclusion

PCA-based approaches can be used to detect, localize and genotype inversions using SNPs. We constructed a new benchmark for validating SNP-based inversion detection methods from publicly-available data. We used this benchmark to perform a more extensive validation of our previously-published inversion analysis framework, and we identified several problematic cases where interpretation can be ambiguous. Lastly, we applied this revised framework to identify 2Rc and 2Rd inversions in Burkina Faso *An. gambiae* and *coluzzii* samples, which were experimentally annotated only while this paper was in press.

Going forward, inversion analysis faces three main challenges. First, the methods evaluated here are not yet developed to the point of being completely automated or “high-throughput.” While progress continues to be made [61], completely automated detection is still out of reach. Secondly, the existing methods are unable to deconvolve cases with multiple mutually-exclusive inversions (e.g., 3R chromosome arm of *D. melanogaster*). Further work needs to look at ways to accurately handle these complicated cases and is already ongoing [60]. Lastly, existing methods require relatively well-assembled, chromosome-length genome assemblies. PCA does not depend on the spatial relationships of SNPs but Manhattan plots resulting from association testing do and significantly improve interpretability. Extending the benchmark and validation presented here to either poorly-assembled genomes or even new reference free (k-mer) detection methods will be useful to the broader research community.

## Supporting information

S1 File Supplemental text. The supplemental text contains additional analysis, including validation using a simulated data set.
(PDF)
