# Supplementary material for: Detecting inversions with PCA in the presence of population structure
Source: PLoS One. 2020 Oct 29;15(10):e0240429. doi: 10.1371/journal.pone.0240429 (PMC7595445; doi:10.1371/journal.pone.0240429)
Supplement: S1 File — The supplemental text contains additional analysis, including validation using a simulated data set. (PDF) [file pone.0240429.s001.pdf]

Supplemental Materials

Principal Component Analysis (PCA) of SNP

Assume that we have N samples with V positions with biallelic variants. Each position has a reference allele and an alternative allele, and at each position, each sample has one of three genotypes (homozygous reference, homozygous alternate, or heterozygous).

We encode the variants as a feature matrix $\mathbf{X}$ with dimensions $N \times 3V$. If sample i has the homozygous reference genotype at position k, then we set $\mathbf{X}_{i,3k+1} = 1$. If sample i has the homozygous alternate genotype at position k, then we set $\mathbf{X}_{i,3k+2} = 1$. If sample i has the heterozygous genotype at position k, then we set $\mathbf{X}_{i,3k+3} = 1$. If the genotype of sample i is unknown at position k, then we do nothing.

There are several advantages for encoding SNPs in this manner. By using one-hot encoding, we can capture situations where a heterozygous mutation may be more informative for a given phenotype than either homozygous mutation. Each feature is independent and can be assigned an appropriate weight (negative or positive) accordingly. Secondly, the one-hot encoding approach allows us to handle multi-allelic sites (e.g., if we were to consider polyploidy data).

Principal component analysis (PCA) of the feature matrix $\mathbf{X}$ produces a $3V \times P$ matrix $\mathbf{W}$ of principal components and a $N \times P$ matrix $\mathbf{T}$ of projected coordinates for the samples such that:

$$\mathbf{T} = \mathbf{X}\mathbf{W}$$

As directly computing PCA would involve computing a $3V \times 3V$ co-variance matrix, we use a randomized PCA method as implemented in Scikit Learn [6]. The whitening flag for PCA was set to `True`, which ensures that the resulting components have unit variance. We use plots of the explained variance ratios to select relevant PCs.

Inferring Inversion Genotypes with K-Means Clustering

Sample karyotypes are inferred by clustering samples using their projected coordinates ($\mathbf{T}$) from PCA. Clustering is performed with the k-means clustering algorithm as implemented in Scikit Learn [6]. We choose the number of clusters K by clustering the samples with 1-6 clusters, plotting the inertia (or sum-of-squared errors), and visually identifying the "elbow" in the plot. We use the default Scikit Learn settings of 10 runs.

The cluster labels can be represented by a $N \times K$ matrix $\mathbf{C}$. Each sample i belongs to one of K clusters, indicated by a value of 1 at position $\mathbf{C}_{i,j}$ where $1 \leq j \leq K$.

Review of Association Testing

Likelihood-ratio tests can be used to test for association between variables with Logistic Regression models. The null hypothesis is that knowing the independent variable does not improve the accuracy of predicting the dependent variable, while the alternative hypothesis is that knowing the value of the independent variable does improve accuracy of predictions because the dependent variable is associated with the independent variable.

In our case, we use a Logistic Regression model, which is appropriate when the independent variable is categorical. The equation for a Logistic Regression model is given by:

$$P(\mathbf{y}_i) = \frac{1}{1 + \exp(-\beta \mathbf{X}_i + \beta_0)} \tag{1}$$

where $\mathbf{y}_i$ is value of the dependent variable for sample i, $\mathbf{X}_i$ is a vector of values for the independent variables for sample i, and β_0 is the intercept.

To evaluate the hypothesis, we compared predictions from a pair of models. The alternative model contains the same independent variables as the null model plus the additional independent variable(s) being

tested against the dependent variable for association. In our case, the null model only contains an intercept (no independent variables) and the alternative model will contain a single independent variable. In cases where the dependent variable is categorical rather than binary, a one-versus-all scheme is used. One model is trained for each category and predicts the probability that the value of the dependent variable is equal to that category.

After fitting the models, we use the models to predict the dependent variable for the samples. From the predictions, we calculate the likelihood for each model. The likelihood for the multinomial Logistic Regression model is given by [1]:

$$L = \prod_{i=1}^{N} \prod_{g} P(\mathbf{y}_{i,g})^{\mathbf{y}_{i,g}} \tag{2}$$

where g is the number of categories the dependent variable can take on.

To perform the likelihood-ratio test, the difference G between the log likelihoods of the two sets of models is calculated by:

$$G = -2(\log L_0 - \log L_\Lambda) \tag{3}$$

where L_0 and L_Λ are the likelihoods of the null and alternative models, respectively.

The p-value for the difference in log likelihoods is calculated using the χ^2 distribution:

$$p = P[\chi^2(df) > G] \tag{4}$$

where df is the difference in the number of degrees of freedom (weights) between the two models.

Scikit Learn was used; we trained the models using Stochastic Gradient Descent (SGD) for 10,000 epochs, the log likelihood, L_2 regularization using the `SGDClassifier` class. All other parameters were left at their defaults. The log likelihoods were calculated with the `log_loss` function (normalize set to `False`). We implement functionality for calculating G and estimating the p-value using Scipy.

PC-SNP Association Tests Adjusted for Missing Data

In [5], we described an approach for localizing inversions using association tests between each SNP and the samples' projected coordinates (T) from PCA. A single association test is performed for each combination of principal component (PC) j and SNP position k, using the coordinate $T_{i,j}$ for sample i along PC j as the independent variable. As the SNPs are encoded as categorical variables, three dependent variables (one for each genotype) are used for each SNP. We employ three Logistic Regression models, one for each genotype, in a one-versus-all scheme.

As the SNPs are the dependent variables, we need a different strategy for handling missing genotypes. We review the method we proposed in [5]. We deterministically up-sample the samples (one copy for each genotype). In particular, if we have M genotypes, we create M copies of each sample. (In our case, $M = 3$ since we are working with biallelic SNPs with three genotypes.) If the genotype is known, the copies have the same genotype as the original. Otherwise, we make the conservative assumption that there is an uninformative (uniform) prior over the genotypes and impute the copies so that there is a one-to-one relationship between the copies and possible genotypes. We also fix the intercept to the class probabilities and did not allow it to change during fitting. Note that unlike the approach for the cluster-SNP association tests, the up-sampled data are used for both fitting the models and in predictions for the calculations of the likelihoods.

Since we increased the number of samples, we need to weight the samples so that the calculated p-values are consistent with the original number of samples. The modified likelihood function is then:

$$L = \prod_{i=1}^{N} \prod_{g} P(\mathbf{y}_{i,g})^{\mathbf{y}_{i,g}/M} \tag{5}$$

Cluster-SNP Association Tests Adjusted for Missing Data

After karyotypes are inferred with clustering, we perform association tests between each SNP and the samples' cluster labels (encoded as a categorical variable). The cluster labels are used as the dependent variables ($\mathbf{y}$), while the genotypes of the SNPs are used as the independent variables ($\mathbf{X}$).

It is common for genotypes in insect SNP data to be unknown (uncalled). We used our approach from [3, 4] to adjust the association tests to avoid bias. For fitting the models, we deterministically up-sampled the samples (one copy for each possible genotype). In particular, if we have M genotypes, we create M copies of each sample. (In our case, $M = 3$ since we are working with biallelic SNPs with three genotypes.) If the genotype is known, the copies have the same genotype as the original. Otherwise, we make the conservative assumption that there is an uninformative (uniform) prior over the genotypes and impute the copies so that there is a one-to-one relationship between the copies and possible genotypes. Additionally, we fix the intercept to the class probabilities and did not allow it to be changed during fitting. For prediction and evaluation of the likelihood, we use the original input data.

Simulated Data

To validate the implementation of our methods, we used invertFREGENE to simulate a data set. We used default parameters for the mutation rate (2.3×10^{-7}), recombination rate (1.25×10^{-7}), proportion of crossovers in recombination hot spots (0.88), length of crossover hot spots (2000), per-base gene-conversation rate (4.5×10^{-8}), and gene-conversion length (500). We simulated 1000 2Mb haploid chromosomes (created from a single founder) in one population and no inversions for 10,000 generations to equilibrate. We introduced an inversion from 0.75 Mb to 1.25 Mb and continued the simulation for another 10,000 generations (or until the inversion frequency reached 50%). We set the MaxFreqOfLostInv parameter to 10% and set the output mode to "sequence" mode. We modify invertFREGENE to output inversion orientations of the haploids. We wrote a custom script in Python to randomly sample haploids without replacement to produce diploid individuals and write a VCF file.

We analyzed the data set using the methods described in the main paper. The explained variance ratios from PCA of the SNPs indicated that 3 PCs explained most of the variance (see Fig. 1a). Three clusters (corresponding to the three inversion genoytpes) were observed when samples were projected onto the first two PCs (see Fig. 1b); clustering with k-means along the first two PCs confirmed three clusters (see Fig. 1d). The third PC appears to cluster the samples as well but not consistently with the inversions (see Fig. 1c). Cluster-SNP association tests (see Fig. 1e) and PC-SNP association tests using PCs 1 and 2 (see Fig. 1f–g) successfully localized the inversion to the expected 0.75 – 1.25 Mbp region. The PC-SNP association tests confirmed that PC 3 was capturing a different effect (see Fig. 1h).

PCA Explained Variance Analysis

To identify which principal components (PCs) should be used in our analyses, we computed and plotted the explained variance ratios for each PC for each data set (see Fig. 2). For some data sets such as the *Drosophila* 2L and 150 *Anopheles* 2L and 2R chromosome arm, some PCs clearly had one or two PCs with elevated explained variance ratios. For other data sets such as the 69 *An. coluzzii* 2R chromosome arm, none of the explained variance ratios of the PCs were clearly elevated. Even in cases with clear enrichment, we found that PCs with non-elevated EVRs still captured inversions (e.g., for *Drosophila* 2L and 2R, the second PCs captured differences between heterozygous samples and other samples – see Table 1).

To be conclusive, we combined the EVA analysis with the PC project plots and PC-SNP association tests (see main paper) to identify which PCs would be most useful for analysis. We attempted to identify sets of PCs which captured in the same inversion. In some cases (e.g, 34 *Anopheles* 2L), an inversion was captured by a single PC. In other cases (e.g., *Drosophila* 2L), two PCs were needed to capture all three genotypes of an inversion.

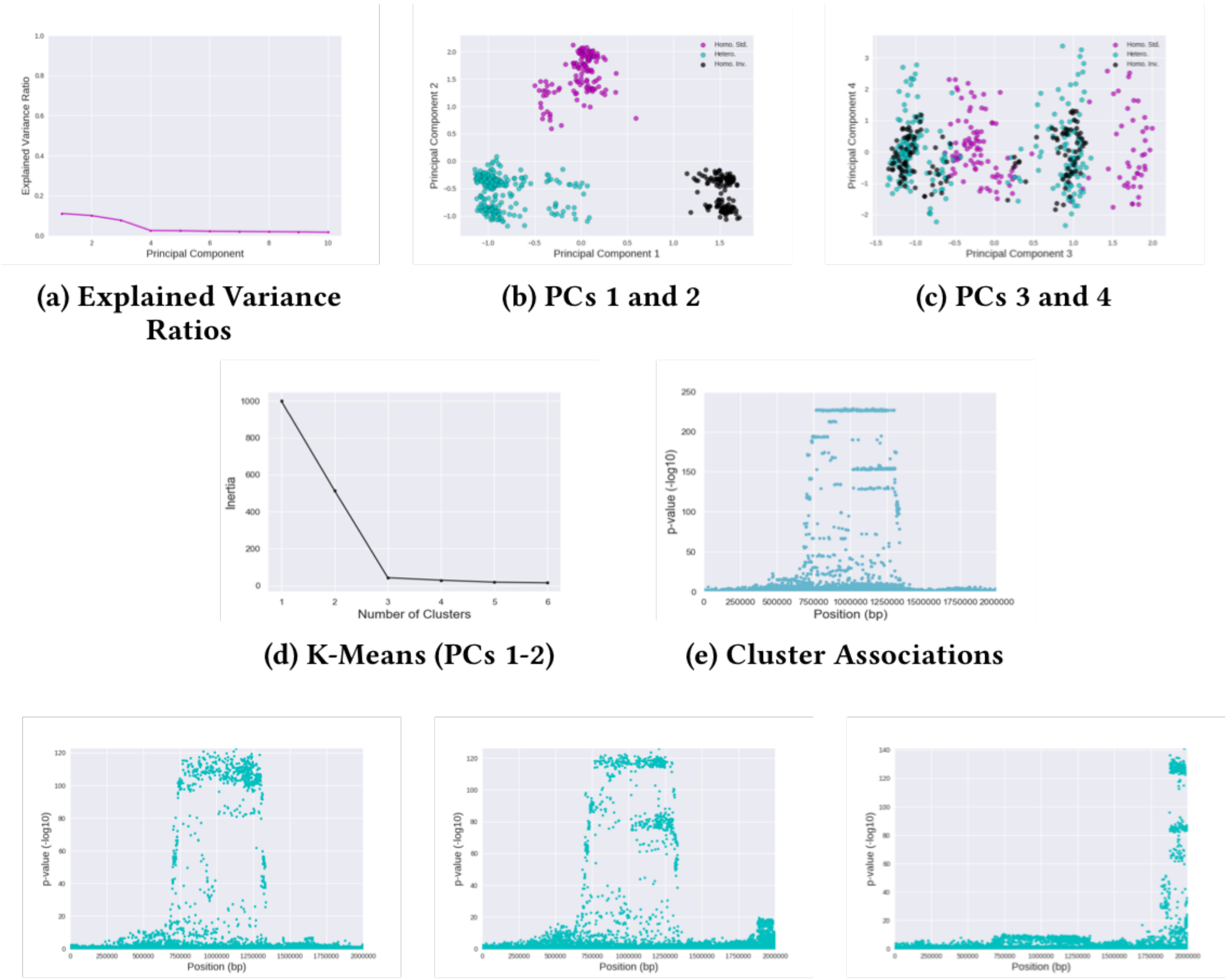

(a) Explained Variance Ratios **(b) PCs 1 and 2** **(c) PCs 3 and 4**

(d) K-Means (PCs 1-2) **(e) Cluster Associations**

(f) PC 1 **(g) PC 2** **(h) PC 3**

Figure 1: **invertFREGENE Simulated Chromosome** Analysis of the 2R chromosome arm of the 150 *Anopheles* samples from Burkina Faso (all samples, 81 *Anopheles gambiae* samples, and 69 *Anopheles coluzzii* samples). (a – c) PCA of samples, clustered with k-means, and colored by cluster. Manhattan plots visualizing p-values from association tests against sample cluster IDs (d – f) and PC coordinates (g – k, one Manhattan plot per PC).

Estimating Number of Clusters

Once a suitable set of PCs was identified for each inversion, we used k-means to cluster the samples. We estimated the number of clusters needed for each data set by running k-means with $k = 1$ to 6, plotting inertia against k, and looking for the “elbow” (see Fig. 3) When an appropriate combination of PCs and value of k, were identified a inertia plot with a clear elbow was observed.

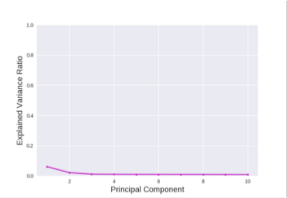

(a) *Drosophila* 2L

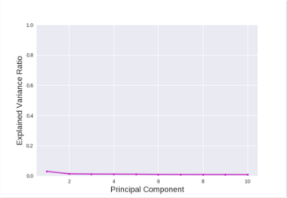

(b) *Drosophila* 2R

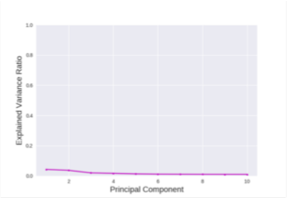

(c) *Drosophila* 3R

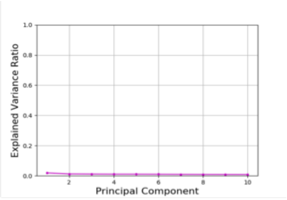

(d) *Drosophila* 3L

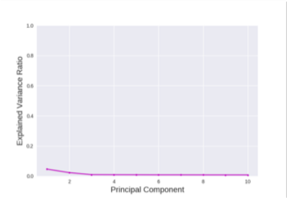

(e) 150 *Anopheles* 2L

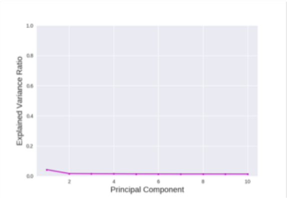

(f) 81 *An. gambiae* 2L

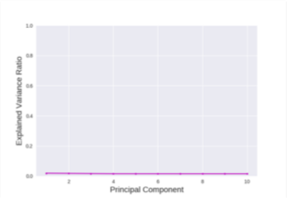

(g) 69 *An. coluzzii* 2L

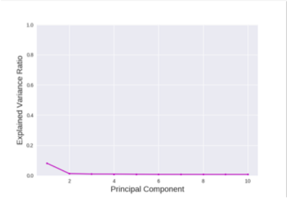

(h) 150 *Anopheles* 2R

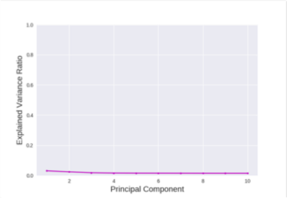

(i) 81 *An. gambiae* 2R

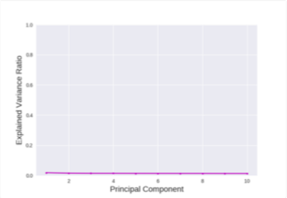

(j) 69 *An. coluzzii* 2R

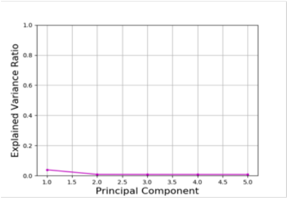

(k) 150 *Anopheles* 3L

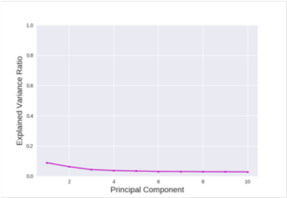

(l) 34 *Anopheles* 2L

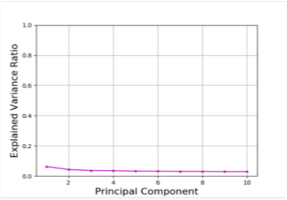

(m) 34 *Anopheles* 3L

Figure 2: **PCA Explained Variance Analysis** PCA was performed with 10 PCs and the explained variance ratios were plotted for each PC. The data sets are for *Drosophila* from the DGRPv2 (a – d), 150 *Anopheles* Burkina Faso samples from the 1000 *Anopheles* Genomes project (e – k), and 34 *Anopheles* samples from Fontaine, et al. (2015) (l – m).

How Does PC Separate Inversion Genotypes?

Huang, et al. reported the experimentally-determined inversion genotypes for each sample in the DGRPv2 [2]. We used these genotype labels in association tests to determine what each PC captured (see Table 1). For the 2L and 2R chromosome arms, the first PCs separated inverted (both homozygous and heterozygous) and non-inverted samples, while the second PCs separated heterozygous samples from the rest. We also observed a similar pattern with our simulated data (see Fig.1). The case where a single PC is able to separate all three genotypes (e.g., 34 *Anopheles* 2L) appears to be a rare occurrence among our data sets.

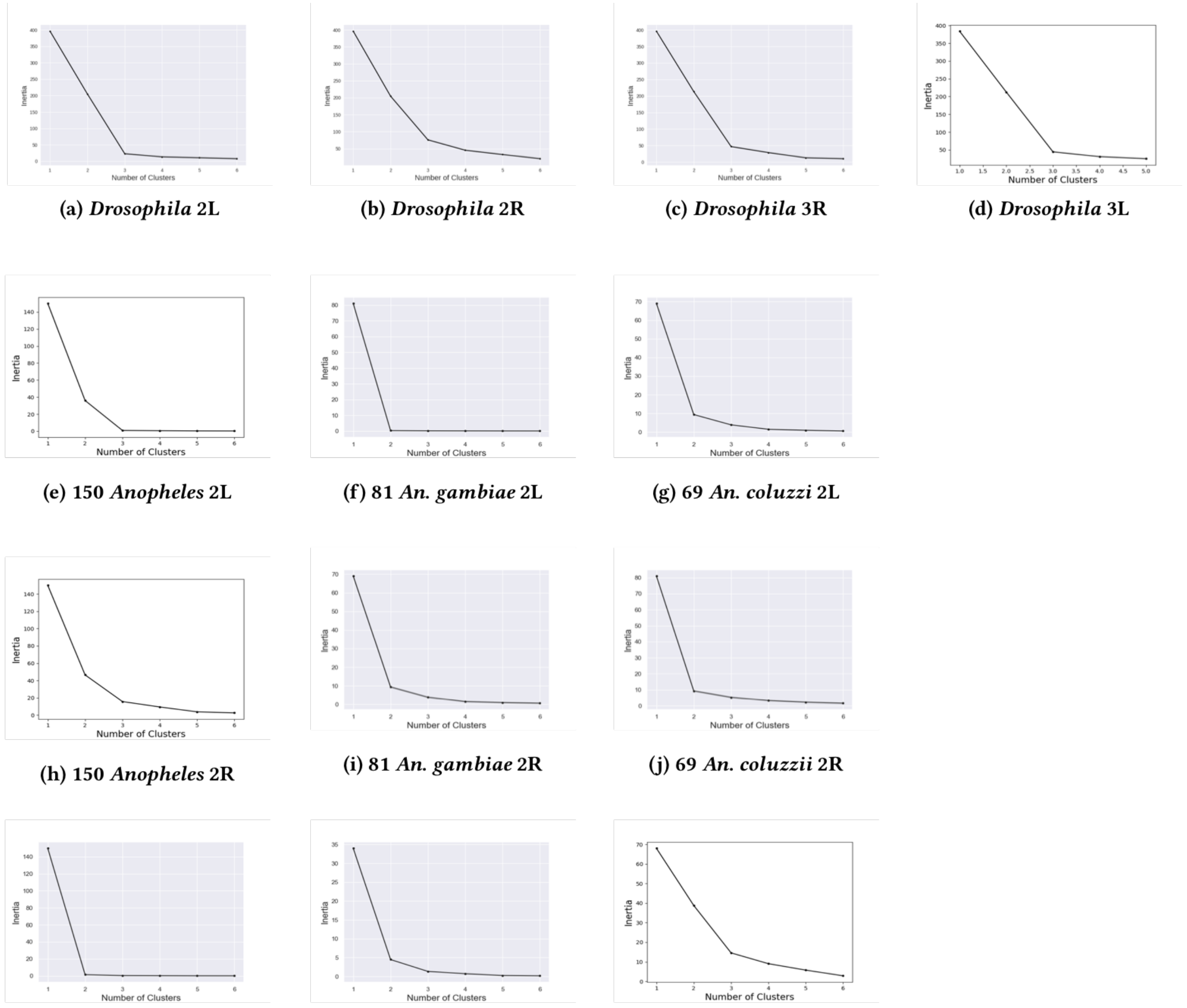

(a) ***Drosophila*** **2L** **(b)** ***Drosophila*** **2R** **(c)** ***Drosophila*** **3R** **(d)** ***Drosophila*** **3L**

(e) 150 ***Anopheles*** **2L** **(f) 81** ***An. gambiae*** **2L** **(g) 69** ***An. coluzzi*** **2L**

(h) 150 ***Anopheles*** **2R** **(i) 81** ***An. gambiae*** **2R** **(j) 69** ***An. coluzzii*** **2R**

(k) 150 ***Anopheles*** **3L (PC 1)** **(l) 34** ***Anopheles*** **2L (PC 1)** **(m) 34** ***Anopheles*** **3L (PCs 1-2)**

Figure 3: **K-Means Cluster Count Analysis** Plots of inertia vs k from k-means clustering. The data sets are for *Drosophila* from the DGRPv2 (a – d), 150 *Anopheles* Burkina Faso samples from the 1000 *Anopheles* Genomes project (e – k), and 34 *Anopheles* samples from Fontaine, et al. (2015) (l – m).

References

[1] D. W. Hosmer Jr., S. Lemeshow, and R. X. Sturdivant. *Applied Logistic Regression.* Wiley, New York, NY, USA, 3 edition, 2013.

[2] W. Huang, A. Massouras, Y. Inoue, J. Peiffer, M. Ràmia, A. M. Tarone, L. Turlapati, T. Zichner, D. Zhu, R. F. Lyman, M. M. Magwire, K. Blankenburg, M. A. Carbone, K. Chang, L. L. Ellis, S. Fernandez, Y. Han, G. Highnam, C. E. Hjelmen, J. R. Jack, M. Javaid, J. Jayaseelan, D. Kalra, S. Lee, L. Lewis, M. Munidasa, F. Ongeri, S. Patel, L. Perales, A. Perez, L. Pu, S. M. Rollmann, R. Ruth, N. Saada, C. Warner, A. Williams, Y.-Q. Wu, A. Yamamoto, Y. Zhang, Y. Zhu, R. R. H. Anholt, J. O. Korbel, D. Mittelman, D. M. Muzny, R. A. Gibbs, A. Barbadilla, J. S. Johnston, E. A. Stone, S. Richards,

Table 1: **Association Tests Between Principal Components and Inversion Karyotypes of *Drosophila* Samples.**

Inversion	Comparison	PC1	PC2
**In(2L)t**	Inverted vs Not	x	
**In(2L)t**	Homo. Inverted vs Rest		
**In(2L)t**	Hetero. vs Rest		x
**In(2R)ns**	Inverted vs Not	x	
**In(2R)ns**	Homo. Inverted vs Rest		
**In(2R)ns**	Hetero. vs Rest		x
**In(3R)P**	Inverted vs Not		
**In(3R)P**	Homo. Inverted vs Rest		
**In(3R)P**	Hetero. vs Rest		
**In(3R)K**	Inverted vs Not		
**In(3R)K**	Homo. Inverted vs Rest		
**In(3R)K**	Hetero. vs Rest	x	x
**In(3R)Mo**	Inverted vs Not		
**In(3R)Mo**	Homo. Inverted vs Rest	x	
**In(3R)Mo**	Hetero. vs Rest		

PCA was performed separately for each chromosome, so the PC columns refer to the PCs for the chromosome of the given inversion.

B. Deplancke, and T. F. C. Mackay. Natural variation in genome architecture among 205 drosophila melanogaster genetic reference panel lines. *Genome Res.*, 24(7):1193–1208, July 2014.

[3] R. J. Nowling and S. J. Emrich. Adjusted likelihood-ratio test for variants with unknown genotypes. In *10th International Conference on Bioinformatics and Computational Biology (BiCOB)*, March 2018.

[4] R. J. Nowling and S. J. Emrich. Adjusted likelihood-ratio test for variants with unknown genotypes. *Journal of Bioinformatics and Computational Biology*, 16(5), 2018.

[5] R. J. Nowling and S. J. Emrich. Detecting chromosomal inversions from dense snps by combining pca and association tests. In *Proceedings of the 2018 ACM International Conference on Bioinformatics, Computational Biology, and Health Informatics*, BCB '18, pages 270–276, New York, NY, USA, 2018. ACM.

[6] F. Pedregosa, G. Varoquaux, A. Gramfort, V. Michel, B. Thirion, O. Grisel, M. Blondel, P. Prettenhofer, R. Weiss, V. Dubourg, et al. Scikit-learn: Machine learning in Python. *Journal of Machine Learning Research*, 12:2825–2830, 2011.
